# Severe headache in primary Sjögren’s syndrome treated with intrathecal rituximab

**DOI:** 10.1002/ccr3.1987

**Published:** 2019-01-17

**Authors:** Anne Bolette Tjensvoll, Maria Boge Lauvsnes, Katrine Brekke Norheim, Roald Omdal

**Affiliations:** ^1^ Department of Neurology Stavanger University Hospital Stavanger Norway; ^2^ Department of Internal Medicine Stavanger University Hospital Stavanger Norway; ^3^ Department of Clinical Science University of Bergen Stavanger Norway

**Keywords:** B cell depletion in CSF, Headache, Primary Sjögren’s syndrome, Systemic lupus erythematosus

## Abstract

A severe and persistent migrainous headache in a patient with primary Sjøgren's syndrome unresponsive to treatment with immunosuppressive drugs, triptans, opioids, and NSAIDs, responded successfully to intrathecal B‐cell depletion with rituximab. We hypothesize that brain‐resident autoreactive B cells were involved in headache pathogenesis and were eliminated by this procedure.

## 1 INTRODUCTION

Severe and treatment‐resistant headache sometimes occurs in patients with systemic lupus erythematosus, so called lupus‐headache.It is defined as a persistent headache that “may be migrainous, but must be nonresponsive to narcotic analgesia”.^1^ We report a case of similar severe and disabling headache in a patient with primary Sjögren's syndrome (pSS) that was successfully treated with intrathecal rituximab.

## 2 CASE HISTORY

A 41‐year‐old woman with a nine‐year history of pSS was admitted to the neurological department due to an intense and disabling migrainous headache that had started a few days earlier. The headache was characterized by a pressing, intense and persistent pain not responding to paracetamol, NSAIDs, triptans, or codeine. At the time of admission, she was on prednisolone 5 mg × 1 due to severe systemic disease activity. She fulfilled the AECG‐criteria for pSS^2^; had subjective dryness of the mouth and eyes, objective reduced tear and saliva production, a positive labial biopsy showing small salivary glands with focus score 2.2, and anti‐SSA and SSB antibodies in high concentrations. IgG in serum was considerably increased 40.5 g/L (5.4‐18.2), and ESR was 114/mm/h, Hgb 11.1 g/dL (11.7‐15.3), all indicating high immunological activity. Extensive investigations by clinical immunologists did not reveal any other disease, especially not lymphoma.

The patient had not suffered from headaches earlier in her life and had no family history of migraine. However, during pregnancy two years prior to the pSS diagnosis, she had experienced visual and sensory aura phenomena without headache. She underwent extensive clinical neurological examinations without revealing any additional abnormal findings. Cerebral MRI with and without gadolinium and intracranial MRI angiography with both arterial and venous series were normal, as was opening pressure on lumbar puncture. Leukocyte and protein content was normal, IgG slightly increased to 0.07 g/L (ref. 0.00‐0.04 g/L), and isoelectric focusing of cerebrospinal fluid (CSF) revealed 6‐7 oligoclonal bands, also present in serum. Borrelia IgG and IgM and Treponema pallidum haemagglutination test were negative. No atypical cells were found, and flow cytometry only showed a reactive pattern. A diagnosis of headache secondary to inflammatory disease was made at the neurological department. She first received weekly intravenous (i.v.) pulses of methylprednisolone 1000 mg and was started on azathioprine 50 mg×2 with moderate response on headaches.

Because of persistent high disease activity, it was decided to try B‐cell depletion with rituximab. She received two initial i.v. doses of 1000 mg 14 days apart, and thereafter 1000 mg every 6 months. In addition, she repeatedly received 1000 mg methylprednisolone every second to fourth week with good but only short‐lasting effect on malaise and headaches (2‐3 days). Nevertheless, over time general disease activity and headache pain gradually declined on this regimen and serum IgG levels normalized. However, headaches tended to increase at the end of intervals between rituximab infusions. Azathioprine was stopped and methotrexate added with some positive effect on the remaining headaches.

Almost four years after the initial headaches and still on rituximab, she suddenly started to have aura episodes with visual and sensory phenomena. Aura episodes went on for days with up to four episodes a day, each lasting for hours. Headaches were bi‐frontotemporal, pressing, intense, persistent and accompanied by nausea and vomiting. On a visual analogue scale 0‐10, she rated the intensity as 10. The headaches again responded briefly to i.v. methylprednisolone that she received regularly up to twice a week. Rituximab was stopped and she consecutively was treated with mycophenolate‐mofetil, tocilizumab, and belimumab to dampen what was assumed to be high immunological activity as cause of the headache. Although she reached partial remission of her pSS on tocilizumab, headaches did not resolve and were persistent with worsening episodes, pressing and sometimes pulsating, accompanied by hypersensitivity to light and sounds, nausea and sometimes vomiting. She avoided activity and preferred to be in bed. She continued to have frequent and multiple aura episodes.

Her headaches, now presenting as chronic migraine with aura, were classified according to the International Classification of Headache Disorders criteria, as headache secondary to an inflammatory disease, namely pSS.^3^ At this time point, the patient was in a paradoxical clinical remission concerning her systemic disease including normal routine hematological and routine laboratory tests and IgG levels, but with severe and disabling headaches. As she had never experienced migraine prior to pSS, a pSS‐associated cerebral process or disturbance not controlled by systemic rituximab was suspected.

It is well known that ectopic germinal centers consisting of T‐, B‐, plasma cells, and follicular dendritic cells may develop in immunological diseases with high immunological activity and take the structural shape of a lymph node.^4^ The presence of these germinal centers in pSS seems to distinguish a certain disease phenotype with high disease activity and increased tendency for malignant transformations to B‐cell lymphomas, and can be seen in 20%‐40% of pSS patients.^5^ Activated T‐ and B cells are able to cross an intact blood‐brain barrier, and we therefore hypothesized that the patient could have brain resident immune cells that were not evident on a recently performed MRI or in the cerebrospinal fluid. These B cells would not have been depleted by rituximab as this drug is prevented from passing the blood‐brain barrier. This could affect/disturb the cerebral homeostasis, thus trigging and maintaining the migrainous headaches. Ectopic germinal centers in the brain have been described in diseases like multiple sclerosis but not in humans with pSS.^6^

To target potential B‐cell migraine generators, she received an intrathecal (IT) injection of 10 mg rituximab in 4 mL 0.9% NaCl after informed consent. Concomitantly, methylprednisolone 500 mg i.v., oral cetirizine 10 mg, and paracetamol 1000 mg were given as premedication. After few days, she responded with reduced frequency of aura episodes and intensity of headaches. After three weeks, headache and aura reappeared and a new IT injection was given with the same positive response. Encouraged by these results, we now gradually increased the dosage and the intervals between injections. Two and a half year after the initial dose she is on a stable regimen consisting of 25 mg rituximab IT every 3 months. On this regimen, she experiences almost total relief of headaches and aura attacks with an endurance of at least three months and is now able to participate in most daily activities. No side effects or complications to the procedure were observed except for transient postpunction headache. As systemic treatment she has continued to receive belimumab 640 mg i.v. and methylprednisolone 1000 mg i.v. monthly.

In conclusion, intrathecal rituximab injections may represent a treatment option for severe headache in patients with autoimmune diseases such as pSS and possibly SLE. The mechanism is unknown but possibly involves depletion of brain resident autoreactive B cells that have survived systemic immunosuppressive therapy.

## CONFLICTS OF INTEREST

None declared.

## AUTHORS’ CONTRIBUTIONS

ABT and RO: designed the study and wrote the first draft. ABT, MBL, KBN, and RO: contributed to the final version of the manuscript, revised it critically for intellectual content, and final sign‐off. All authors also examined and were engaged in clinical follow‐up and collecting data of the patient. RO provided overall guidance and support in all responsibilities.
